# Association between women’s empowerment and demand for family planning satisfied among Christians and Muslims in multireligious African countries

**DOI:** 10.1136/bmjgh-2023-013651

**Published:** 2024-05-09

**Authors:** Franciele Hellwig, Yohannes Wado, Aluisio J D Barros

**Affiliations:** 1 International Center for Equity in Health, Federal University of Pelotas, Pelotas, Brazil; 2 Posgraduate Program of Epidemiology, Federal University of Pelotas, Pelotas, Brazil; 3 African Population and Health Research Center, Nairobi, Kenya

**Keywords:** epidemiology, public health, cross-sectional survey

## Abstract

**Background:**

Although the levels of demand for family planning satisfied (DFPS) have increased in many countries, cultural norms remain a significant barrier in low- and middle-income countries. In the context of multireligious African countries, our objective was to investigate intersectional inequalities in DFPS by modern or traditional contraceptives according to religion and women’s empowerment.

**Methods:**

Analyses were based on Demographic and Health Surveys carried out between 2010 and 2021 in African countries. Countries with at least 10% of Muslims and Christians were selected to analyse inequalities in family planning. The religious groups were characterised by wealth, area of residence, women’s age and women’s empowerment. The mean level of empowerment was estimated for each religious group, and multilevel Poisson regression was used to assess whether DFPS varied based on the level of women’s empowerment among Muslims and Christians.

**Results:**

Our study sample of 14 countries comprised 35% of Muslim and 61% of Christian women. Christians had higher levels of empowerment across all three domains compared with Muslims and women with no/other religion. DFPS was also higher among Christians (57%) than among Muslims (36%). Pooled analysis indicated a consistent association between DFPS and women’s empowerment, with higher prevalence ratios among Muslims than Christians, especially in the decision-making domain.

**Conclusions:**

The gap between Muslims and Christians in DFPS significantly reduced as the level of empowerment increased. It highlights the importance of understanding and addressing cultural factors sensibly and respectfully to satisfy the demand for family planning services.

**WHAT IS ALREADY KNOWN ON THIS TOPIC**

- Prior studies have demonstrated that there is a complex and variable relationship between religion and family planning beliefs in sub-Saharan Africa.
- Higher fertility and lower use of contraceptives among Muslims than among Christians were documented in several African countries.
- The literature on the relationship between women’s empowerment and contraceptive use is mixed, with some studies identifying null associations and others finding positive associations.
- A qualitative study conducted in Tanzania has identified that both religion and gender dynamics influence family planning practices.

**WHAT THIS STUDY ADDS**

- Our study builds on the prior literature by using data from 148 989 women to investigate intersectional inequalities in demand for family planning satisfied (DFPS) by religion and women’s empowerment across multiple countries.
- While Muslim was the group with lower levels of women’s empowerment in the three survey-based women's empowerment index (SWPER) domains, African Christians were on average more empowered than the average of women from all low- and middle-income countries.
- Lower levels of DFPS were also identified among Muslim women; however, the coverage increased significantly with their empowerment. With the highly empowered Muslims having higher DFPS with modern methods than the highly empowered Christians.
- Among the SWPER domains, stronger effects were found in the decision-making domain.
- Higher pooled prevalence ratios were identified when considering traditional methods instead of modern contraceptives.

**HOW THIS STUDY MIGHT AFFECT RESEARCH, PRACTICE OR POLICY**

- This study can help researchers, policymakers and policy managers to better understand how social norms affect the use of family planning services.
- While religious beliefs can be a challenge to family planning, promoting gender equity and empowering women can help to mitigate these challenges and improve access to reproductive healthcare services for women.

## Introduction

Although considerable strides have been made to increase levels of the demand for family planning satisfied (DFPS) with modern methods in low- and middle-income countries (LMICs),^1 2^ there are still persistently low levels of coverage in certain countries and among certain groups of women, such as the poorest, youngest age groups, Muslims and those with lower levels of education.^3^ Increasing family planning use is especially challenging in sub-Saharan Africa, where a significant portion of the population lives in extreme poverty, and health services fail to reach many women.^4–6^ Nevertheless, the influence of cultural factors such as gender inequality, early marriage, fertility expectations and negative attitudes towards family planning practices is progressively assuming a central role.^3 7–9^

Gender inequality is crucial in shaping women’s access to and utilisation of reproductive health services. This is influenced by various factors, including women’s limited participation in household decisions, restricted decision-making capabilities related to their fertility, lack of mobility, financial constraints and the occurrence of domestic violence.^7 10 11^ While evidence on the impact of women’s empowerment on family planning utilisation remains inconclusive due to methodological limitations, studies generally suggest that higher levels of empowerment, particularly decision-making and freedom of movement, are associated with increased family planning utilisation.^12–15^

The relationship between family planning and women’s empowerment is often intertwined with societal and personal values,^16^ religion being one of them. Evidence suggests religious leaders can influence individual behaviour, including their reproductive health choices.^8 17^ Condemnation of modern contraception due to religious beliefs has been linked to lower family planning use.^17–20^ However, religions are not similar in their views on contraceptive usage, and they can affect practices differently.^17 21^ Also, religious teachings affect individual choices differently across affiliations and social contexts. Some studies showed that Christian women tend to present higher family planning utilisation than Muslims.^3 22^

The relationship between women’s empowerment, religion and family planning utilisation is a complex subject that has not been sufficiently explored in the literature. This study explores the independent effects of women’s empowerment and religion on DFPS and whether the intersection of these effects is relevant. The study used data from African countries where Muslim and Christian women are found in large enough proportions and focused on family planning with modern and traditional contraceptives.

## Materials and methods

### Data sources

We used publicly available data from the most recent Demographic and Health Surveys (DHS) carried out from 2010 to 2021. In the 14 African countries with available data on religion, women’s empowerment and DFPS, women were classified into Muslim, Christian or other (other faiths or unaffiliated) according to their report. The analyses included only those countries where the proportion of Christian women and Muslim women individually constituted at least 10% of the total population. The analyses were based on currently partnered women aged 15–49.

### Outcome

Our main outcome was DFPS, defined as the proportion of women in need of contraception who were using (or whose partner was using) a contraceptive method. A woman was considered in need of contraception if she was fecund and did not want to become pregnant within the next 2 years or was unsure about whether or when she wanted to become pregnant. Pregnant women with a mistimed or unintended pregnancy were also considered in need of contraception.

DFPS was explored according to the type of contraceptive method used, modern or traditional. Modern contraceptives were defined as medical procedures or technological products,^23^ including oral contraceptive pills, injections, male and female condoms, diaphragms, spermicidal agents, emergency contraception, intrauterine devices, implants and sterilisation (female or male). Traditional methods included all calendar-based methods, lactational amenorrhea and withdrawal.

### Stratifiers and descriptive variables

Women’s empowerment was measured using the SWPER Global,^24 25^ an individual-level indicator estimated for women aged 15–49 who are married or in a union. The SWPER was derived using principal component analyses based on 14 DHS questions covering three domains of women’s empowerment: (1) social independence, related to access to information, education and age of marriage and first birth; (2) decision-making, related to making decisions on important household matters and (3) attitude towards violence, related to how much the woman rejects domestic violence against the wife. The resulting scores were standardised so that positive values represent above-average levels of empowerment across LMICs used to derive the indicator, while negative values represent the opposite.^24^ The zero value represents average empowerment across the LMICs used to derive the indicator.^24^ Based on the continuous SWPER scores, each domain was categorised into low, medium and high levels of empowerment based on approximate tertiles.

### Statistical analyses

We conducted pooled individual-level analyses across the selected countries using multilevel Poisson regression to evaluate the overall association between religion, women’s empowerment and DFPS by traditional and modern methods. The multilevel model was structured in two levels: women at level one and countries at level two. The second level was included only to consider the within-country correlation, the model did not include contextual factors in the adjustment. The interaction between women’s empowerment and religion was investigated in the regression models and the differences in the association between Christian and Muslim women were assessed using separate religion-specific regression models. Sensitivity analyses were tested by individual wealth and by country’s majority religion. In the first sensitivity analysis, we restricted the sample to the 40% poorest and 40% wealthiest women, regardless of their country. In the second, we selected only countries with more than 60% of the population reporting to be followers of one religion. Muslim-majority countries included in the restricted sample were Burkina Faso, Guinea and Sierra Leone. Christian-majority countries were Ethiopia, Ghana, Liberia, Malawi, Mozambique and Uganda.

All analyses were performed using Stata software V.18.0 (StataCorp, College Station, Texas, USA) and adjusted for the sample design, including sample weights, clusters and strata. All analyses relied on publicly available anonymised databases. Institutions and national agencies in each country obtained ethics approval for the surveys.

## Results

### Description of the selected countries

A total of 14 countries that fulfilled the inclusion criteria were included in the analysis. 10 countries were from West and Central Africa, and four were from Eastern and Southern Africa. Christians were the majority in eight countries (Malawi, Uganda, Liberia, Ghana, Togo, Mozambique, Benin and Ethiopia), while Muslims were the majority in five (Guinea, Sierra Leone, Burkina Faso, Nigeria and Chad). In Côte d’Ivoire, Christians represented 46% of the population, while Muslims accounted for 40%. The other or unaffiliated religious groups were generally small, except in Togo (26%), Mozambique (21%), Benin (16%) and Côte d’Ivoire (16%) (table 1).

**Table 1:** Distribution of religious groups and demand for family planning satisfied (DFPS) in the 14 study countries

Country	Religion			DFPS (%)			N (unweighted)
	Muslim (%)	Christian (%)	Other/unaffiliated (%)	Traditional	Modern	Any	
Benin (2017)	27.8	55.9	16.3	6.7	24.9	31.6	11 170
Burkina Faso (2010)	61.7	31.8	6.5	3.0	36.6	39.6	13 392
Chad (2014)	45.8	51.1	3.1	5.4	14.0	19.4	13 439
Côte d'Ivoire (2011)	40.9	43.4	15.7	10.8	25.6	36.4	6453
Ethiopia (2016)	30.3	68.4	1.3	1.4	60.2	61.6	9824
Ghana (2014)	14.6	79.9	5.4	7.3	38.4	45.6	5456
Guinea (2018)	84.8	12.2	3.0	11.4	19.7	31.1	7812
Liberia (2019)	12.7	85.6	1.7	1.5	40.5	42.0	4654
Malawi (2015)	11.6	87.8	0.7	1.5	74.4	75.9	15 952
Mozambique (2015)	18.7	60.4	20.9	4.9	48.0	52.9	4363
Nigeria (2018)	45.9	53.7	0.4	14.6	30.4	45.0	28 888
Sierra Leone (2019)	77.4	22.6	0.0	0.9	44.9	45.8	9837
Togo (2013)	16.1	58.0	25.9	4.3	32.2	36.5	6360
Uganda (2016)	13.7	85.3	0.9	7.5	49.7	57.1	11 379

DFPS ranged from 19% in Chad to 76% in Malawi, with the modern method being the most commonly used in all countries. DFPS with traditional methods was higher than 10% only in Nigeria (15%), Guinea (11%) and Côte d’Ivoire (11%) (table 1).

Table 2 describes the religious groups according to key characteristics. No meaningful difference in terms of women’s age was observed between the three religious groups. Muslims and Christians exhibited comparable distributions in terms of area of residence and wealth quintiles. Women with other religions or unaffiliated were mostly rural (76%) and poorer (60% were from the two poorest wealth quintiles). Regarding women’s empowerment, Christians presented higher levels of empowerment across all three SWPER domains, especially in the decision-making domain, where 55% of Christians were in a high level of empowerment while only 36% of Muslims were highly empowered.

**Table 2:** Description of the main religious groups in terms of wealth, area of residence, women’s age and women’s empowerment

	Religion			P value*
	Muslims	Christians	Other/unaffiliated	
Area of residence				<0.001
Urban	39.2	35.1	24.3	
Rural	60.8	64.9	75.7	
Wealth				<0.001
Poorest	20.1	17.6	35.6	
Poorer	18.4	19.7	23.9	
Middle	19.2	20.2	18.3	
Wealthier	20.6	20.3	13.0	
Wealthiest	21.7	22.2	9.1	
Women’s age (years)				<0.001
15–19	5.0	4.6	5.3	
20–34	57.1	58.7	56.3	
35–49	37.9	36.6	38.4	
*Women’s empowerment*				
Social independence				<0.001
Low	44.3	30.4	47.9	
Middle	30.6	39.3	34.8	
High	25.1	30.3	17.3	
Attitude to violence				<0.001
Low	28.1	21.5	26.8	
Middle	18.3	19.0	21.3	
High	53.6	59.5	51.8	
Decision-making				<0.001
Low	32.4	13.0	23.0	
Middle	32.0	31.7	38.1	
High	35.5	55.4	38.9	

*Pearson χ^2^.

**Figure 1:**
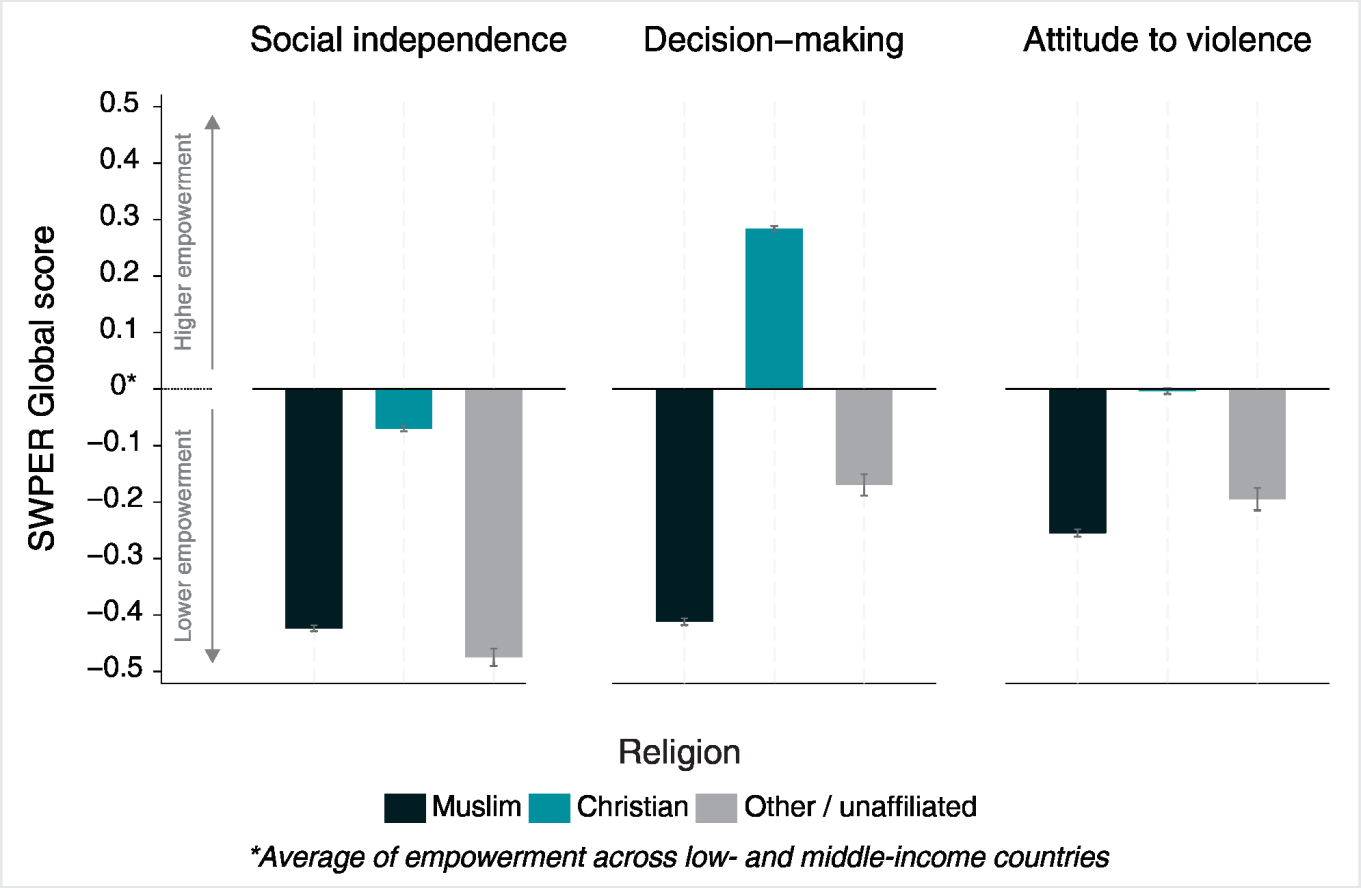
Mean level of women’s empowerment (measured by the survey-based women's empowerment index (SWPER Global)) according to religion.

The mean level of empowerment according to each SWPER domain and religion is presented in figure 1. Muslims had lower levels of empowerment than Christians in all three domains, with negative values in all the SWPER Global domains, indicating a level of empowerment below the global average for LMICs. Christians had higher levels of empowerment than the global average in the decision-making domain and levels close to the average in attitude to violence and social independence.

### DFPS according to women’s empowerment and religion

**Figure 2:**
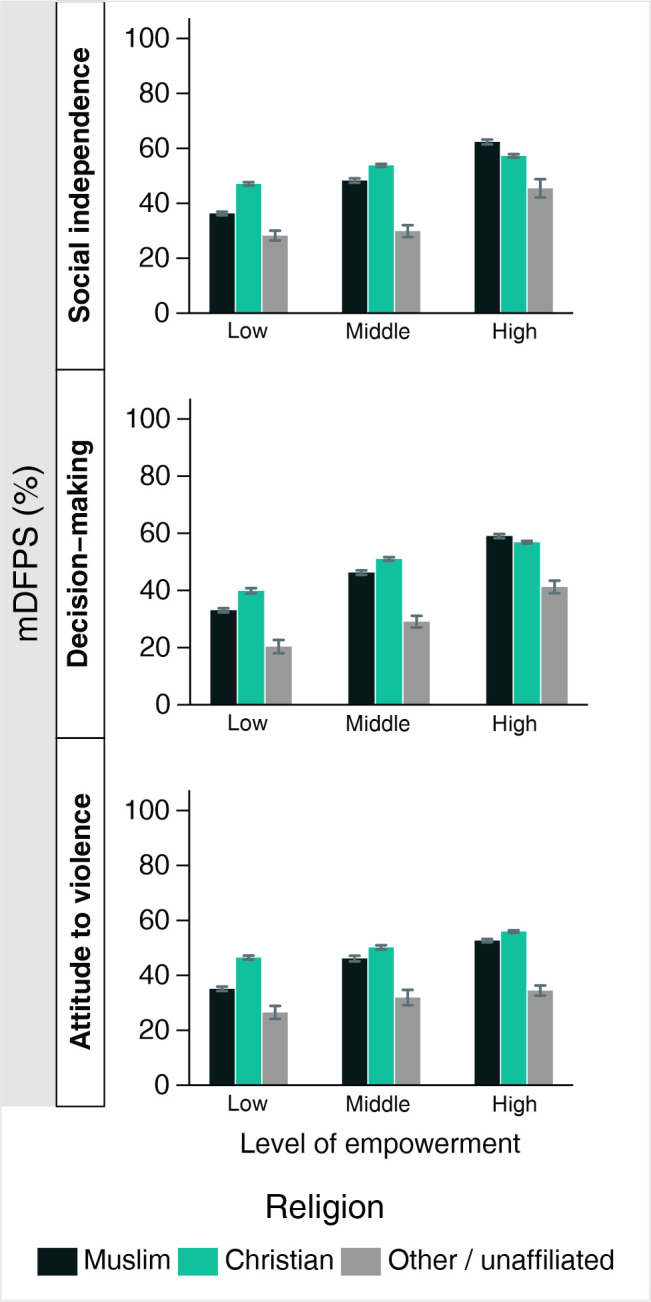
Demand for family planning satisfied by modern methods (mDFPS) according to religion and women’s empowerment.

Figure 2 presents the demand for family planning satisfied by modern methods (mDFPS) for each SWPER domain and according to each religious group. There are marked differences according to religion, particularly among women with lower levels of empowerment. The largest gaps were identified in the domains of social independence and attitude to violence, where mDFPS was 11 percentage points higher among low empowered Christians in comparison with low empowered Muslims. The pattern was reversed among highly empowered women, where Muslims had higher mDFPS compared with Christians. The largest difference was in social independence, with highly empowered Muslims having mDFPS 5 percentage points higher than the highly empowered Christians. Irrespective of the level of empowerment and SWPER domain, women with other or no religion had the lowest mDFPS. Smaller disparities were observed when considering the demand for family planning satified by traditional contraceptive methods (tDFPS). Results can be found in online supplemental material.

10.1136/bmjgh-2023-013651.supp1 Supplementary data

### DFPS and the intersection between religion and women’s empowerment

**Figure 3:**
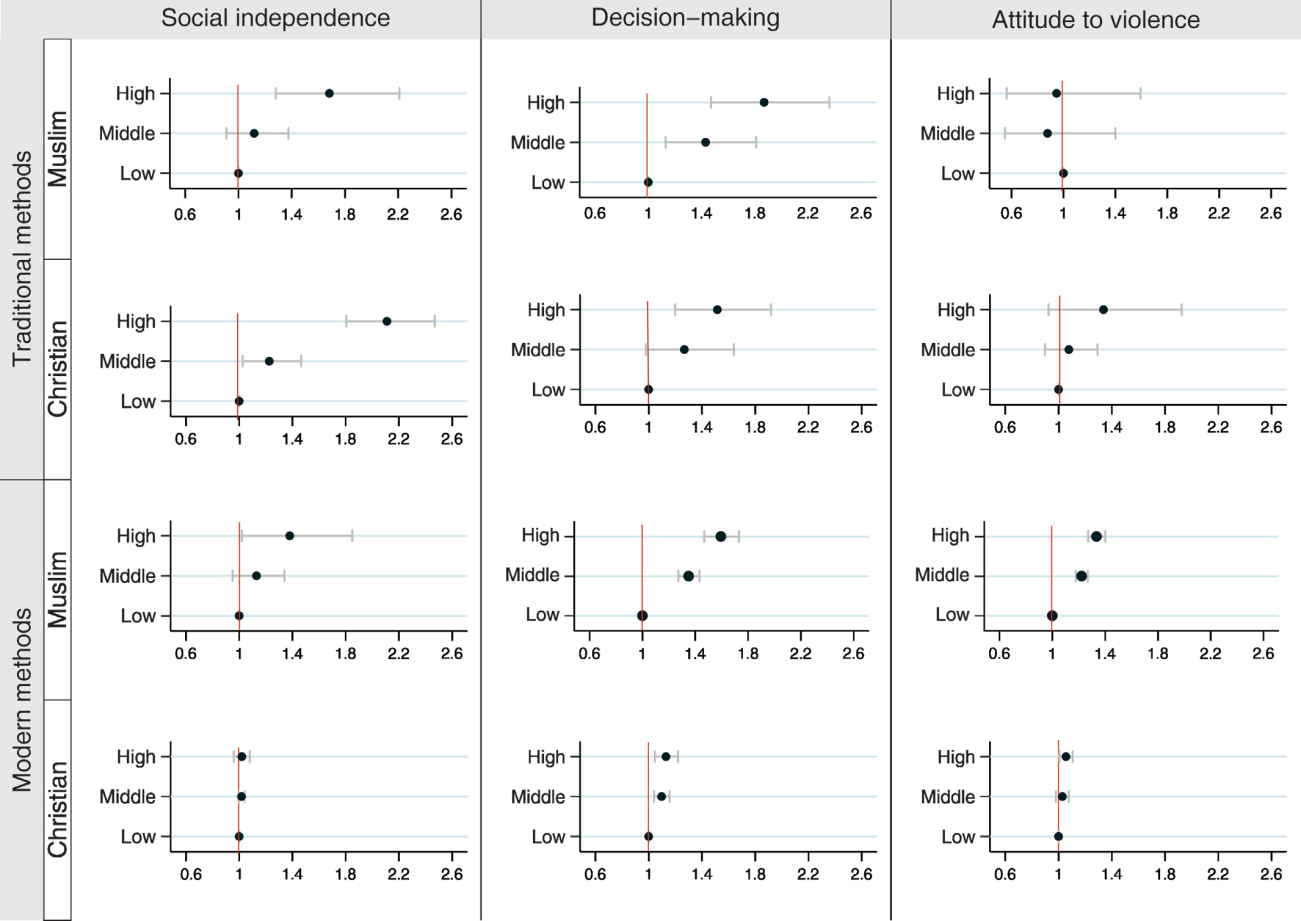
Pooled prevalence ratios for demand for family planning satisfied according to type of method, religion and level of women’s empowerment. All the interaction tests had p<0.001.

Figure 3 shows the prevalence ratios comparing high and middle levels of empowerment with low levels in terms of DFPS, for Muslims and Christians separately. We found a consistent association between DFPS and religion, with larger prevalence ratios among Muslim women. The prevalence ratios, along with their 95% CIs and interaction p values, are presented in online supplemental table 1.

We identified a significant relationship between women’s empowerment and DFPS in terms of decision-making power. Regardless of religion or type of contraceptive used, we found that high levels of empowerment were associated with higher DFPS. The highest prevalence ratio was observed among highly empowered Muslims, with their tDFPS being 1.9 times higher than that of the low empowered women. When modern contraceptive methods were considered, the prevalence ratios in this domain decreased significantly among Christians. Among highly empowered Christians, the tDFPS was 50% higher than among the low empowered group, while mDFPS was only 10%. In contrast, the prevalence ratios remained high among Muslims. The mDFPS among highly empowered Muslims was 50% higher compared with the low empowered women.

In the domain of social independence, larger prevalence ratios were observed when considering the use of traditional methods, with Christians exhibiting the highest prevalence ratios. High empowerment among Christians and Muslims was associated with tDFPS 2.1 and 1.7 times higher in comparison with low empowerment. When modern methods were considered, no difference in terms of empowerment level was observed among Christians. Among Muslims, mDFPS was 13% and 38% higher among Muslims in the middle and high levels of empowerment, respectively, than among low empowered Muslims.

No difference was found in tDFPS in the attitude to violence domain between levels of empowerment nor between Muslims and Christians. Among Christians, we also observed no difference in mDFPS across different levels of empowerment. Nevertheless, a significant association between mDFPS and women’s empowerment in terms of attitude towards violence was identified among Muslims. Muslim women in the middle and high levels of empowerment presented levels of mDFPS 20% and 29% higher, respectively, than those of the low empowered group.

Considering the heterogeneity in our sample, we carried out two types of sensitivity analysis, restricting our sample according to individual wealth and according to country’s majority religion. The results indicated only a marginal difference between the subgroups and the whole sample, which does not appear to be systematic. Results can be found in online supplemental material.

## Discussion

In this multicountry study, we found significant intersectional inequalities in DFPS regarding religion and women’s empowerment. African Christians were more empowered than women from other religious groups in Africa and even more empowered than the average women in LMICs. Additionally, levels of DFPS were higher among Christians than among other religious groups. The relationship between women’s empowerment and DFPS was stronger among Muslims than Christians, leading to a narrowing gap in DFPS among Muslims and Christians with the increase in the empowerment levels. Notably, highly empowered Muslims presented higher levels of mDFPS than highly empowered Christians.

Our findings align with previous studies that have shown higher use of contraceptives among Christians compared with Muslims.^26–28^ Islam allows for different interpretations of the Quran and the Sunnah, leading to variations in the acceptance of contraception.^18 29 30^ While some conservative Muslims argue against any form of contraception, other schools permit fertility regulation for birth spacing.^18 30^ Previous studies investigating family planning barriers among African Muslims have identified that due to Islamic beliefs mandating women to grant their husbands unrestricted access to their bodies and the potential resulting pregnancies,^19 20^ their concerns about family planning practices goes beyond fate for the afterlife, as they also fear domestic violence if their husbands suspect them of seeking contraceptives.^20^ Additionally, the perspectives of Muslims on family planning are also shaped by political contexts.^8 20 31^ While people from different religious backgrounds may also question the relevance and origins of birth control ideas, Muslims, in general, tend to be more reluctant to embrace contraception due to their belief that international family planning efforts are politically motivated.^8 31^ In agreement, increases in the use of contraception have been documented in countries where family planning policies were implemented without any political connotation.^31^

Christian attitudes towards family planning vary significantly depending on the denomination. The three major denominations of Christianity are Roman Catholicism, Eastern Orthodoxy and Protestantism. Catholicism sees marital sex as a means of procreation and marital unit. These two aspects are seen as inextricably linked. Thus, refusing one will inevitably undermine the other.^32^ Within Catholicism, periodic abstinence methods are allowed for birth spacing in specific cases, such as when a pregnancy would be associated with health or socioeconomic risks.^18 21 33^ Analogous to Catholicism, Eastern Orthodoxy also sees marital sex as a mean of procreation and marital unit but it differentiates between types of contraceptives, accepting condoms and withdrawal but rejecting hormonal contraceptives and sterilisation.^18 21^ While some more conservative Protestant denominations are against contraception, Protestantism, in general, takes a more flexible approach to secular issues, including an acceptance of modern contraceptive use.^18 21^

The impact of religion on family planning is influenced by the level of flexibility exhibited by religious representatives and women in navigating religious dogmas. The varying stances taken by representatives of different religions on contraception have been argued to reflect their respective willingness to preserve their positions in society.^31^ Evidence suggests that women can reconcile their secular views with religious beliefs, while religious institutions may serve as valuable sources of social power and may offer a platform for discussions on gender roles and healthcare.^33–35^ Additionally, within Christianity, there are several organisations promoting women’s empowerment in Africa. These organisations have been implementing a number of actions, including promotion of academic study for women, provision of tailoring classes, distribution of loans to allow vulnerable women to start small businesses, creation of study and support groups and seminars on relevant issues such as domestic violence and youth empowerment.^36–38^ In line with this, our findings indicate that Christian African women were not only more empowered than African women following other religions or unaffiliated, as their level of empowerment in the decision-making domain surpasses the average level observed among women across LMICs from all regions. This is a notable finding given the generally poorer socioeconomic conditions in most of the African countries.

The literature consistently underscores the significance of engaging religious leaders in promoting family planning. Consequently, there has been a shift among religious leaders towards increased openness in endorsing and advocating for family planning initiatives, especially by sharing messages about birth spacing through the use of traditional contraceptive methods.^39–41^ However, it is essential to recognise that the impact of religion on contraceptive use is influenced by a complex interplay of socioeconomic and cultural factors.^28 42^ Factors such as education and gender equality not only affect women’s access to and utilisation of modern contraceptives but also their ability to properly use traditional contraceptives, which require knowledge of the ovulatory cycle and negotiation within intimate relationships.^12–14 43 44^

By examining the intersectional inequalities in the use of traditional and modern methods based on three domains of women’s empowerment, we found larger gaps between low-empowered and high-empowered women in demand satisfied. The stronger association was identified among DFPS by traditional methods and the social independence domain. Variables within this domain, such as women’s education, age at first birth and first cohabitation, and spousal differences in age and education, have been associated with contraceptive use.^3 45–47^ This association may be influenced by a range of factors related to both the health system and sociocultural norms. While most women in our study had their DFPS by modern methods, traditional methods may serve as practical alternatives for those seeking to limit or space their births when access to modern contraceptives is limited or their partner does not approve of contraceptive practices. Unlike several modern contraceptives that require minimal effort and can even be hidden from the husbands, the traditional methods, mostly represented by withdrawal and fertility-awareness methods, require a deeper knowledge of women’s reproductive cycle, discipline and the power to negotiate sexual matters with their husbands. More than in relation to modern contraceptives, the women’s level of empowerment is a central factor for their use of traditional contraceptives. In other ways, in contexts where specific contraceptive methods are strongly endorsed, more empowered women may overcome these norms to align their choices with specific preferences, which may include opting for traditional methods. These findings underscore the complexity of the relationship between women’s empowerment and contraceptive choices, warranting further exploration.

When examining the utilisation of modern contraceptives, differences were found across all domains, with the decision-making domain yielding more significant results than the other domains. The SWPER decision-making domain includes the woman’s freedom to decide on visiting friends or relatives, on significant household purchases and on her own healthcare. The influence of women’s decision-making power on contraceptive use has been previously explored, with joint decision-making being associated with higher contraceptive usage.^48–51^

Our findings of a narrowing gap in DFPS among Muslims and Christians with the increase in the empowerment levels indicate that the effects of women’s empowerment on DFPS were significantly higher among Muslims, with highly empowered Muslims showing the highest levels of DFPS. This finding shows that religion is not a unidimensional force that singularly oppresses women. Instead, the strength of religious practices and beliefs and how they interact with other factors will determine if and to which extent the religion will interfere with women’s daily choices and self-perception.^35^ Additionally, there is evidence that the negative impact of the Islamic religion on gender equality is weakened by women’s education.^35^ Indicating that sociocognitive empowerment may empower women to deviate from extremist religious interpretations.^35^ Furthermore, countries with more conservative Muslim societies often display less openness to the impacts of modernisation.^52^ Considering that there is a larger educational gender gap among Muslims compared with followers of other religions,^52^ highly empowered Muslims likely represent a specific group characterised by exceptional education levels and a greater willingness to embrace new ideas and technologies. Further exploration is required to better understand the pathways through which women’s empowerment operates within Muslim communities.

Evidence of the relationship between women’s empowerment and women’s reproductive health was previously summarised in the scientific literature.^12–15^ The various studies identified have defined women’s empowerment differently, leading to diverse findings depending on the study population, context and the specific domain considered. More consistent effects were found when measuring women’s empowerment through a composite score or index.^12–15^ While there are numerous composite measures to assess women’s empowerment,^53^ the women’s empowerment index used in this study demonstrated high cross-country consistency, capturing meaningful variations in women’s empowerment levels across diverse cultural and socioeconomic contexts and enhancing the robustness of our findings.^24 25^ As the SWPER incorporates several factors strongly associated with family planning coverage, including wealth, area of residence and women’s education, we opted to not adjust our analyses for these relevant variables.

It is important to acknowledge the limitations of this study. One such limitation is the lack of consideration for the heterogeneity within Muslims and Christians, as well as their varying degrees of adherence to religious dogmas. The unavailability of such detailed information in national health surveys limits the depth of our assessment.

Despite these limitations, this study provides valuable insights into the complex relationship between religion, women’s empowerment and the DFPS. To fully comprehend the influence of religion on family planning, it is essential to consider the cultural, historical, political and sociodemographic contexts within which it operates. These contextual factors shape how religious groups position themselves in relation to cultural and technological advancements. Moreover, it is important to recognise that religion offers individuals a broad and transcendental perspective to navigate life, while public policies and strategies often adopt a narrow perspective based on their specific objectives. The challenge of developing effective evidence-based health policies lies in striking a balance between the need for a broader perspective, considering these diverse contextual factors, and the inherent limitations imposed by technical feasibility and the availability of financial and non-financial resources for implementation. Investing in studies that delve into these intricate relationships and involving stakeholders in all stages of the policy development process can lead to a more nuanced comprehension of the sociocultural dynamics and facilitate the development of more impactful policies.

## Conclusion

This study demonstrated a significant association between women’s empowerment and DFPS among Muslim women. It also showed that the disparity between Muslims and Christians in DFPS significantly reduced as the level of empowerment increased. By presenting a comprehensive portrayal of this intersectional inequality, our findings could enhance comprehension of the interplay between women’s empowerment, religion and family planning. It emphasises the critical need to approach cultural factors with sensitivity and respect to effectively meet the demand for family planning services. Policymakers and healthcare providers should acknowledge the potential impacts of gender roles and religion on a couple’s decision to use contraception and be knowledgeable about the diverse range of family planning options available. By doing so, they can effectively navigate and respond to these cultural and social circumstances in the most optimal manner, ensuring that individuals’ reproductive health needs are met with sensitivity and respectability.

## Data Availability

All data relevant to the study are included in the article or uploaded as supplementary information.
