# Supplementary material for: Association between women’s empowerment and demand for family planning satisfied among Christians and Muslims in multireligious African countries
Source: BMJ Glob Health. 2024 May 9;9(5):e013651. doi: 10.1136/bmjgh-2023-013651 (PMC11085792; doi:10.1136/bmjgh-2023-013651)
Supplement: Supplementary data [file bmjgh-2023-013651supp001.pdf]

Supplementary material

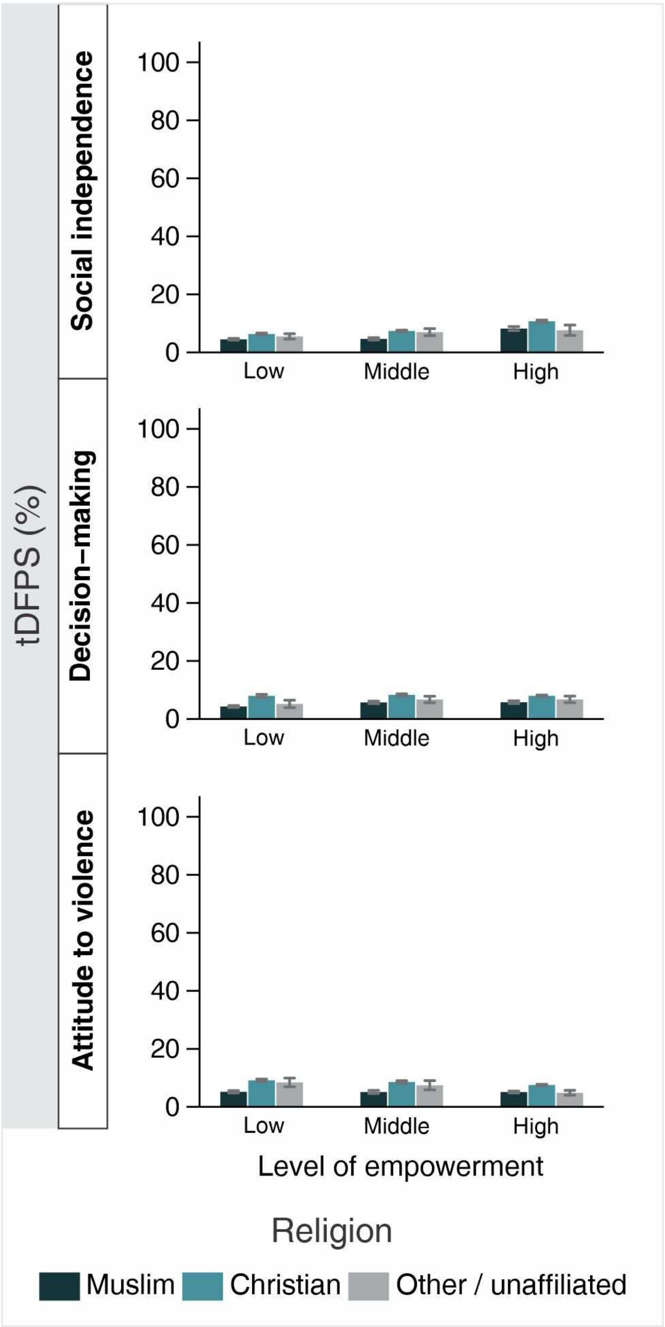

Supplementary Figure 1. Demand for family planning satisfied by traditional methods according to religion and women's empowerment.

Supplementary Table 1. Demand for family planning satisfied according to religion, type of method, and women's empowerment in the selected countries.

	Muslim		Christian		Interaction p value
	IRR	95% CI	IRR	95% CI	
**Traditional methods**					
Social independence					0.000
Low	1.00		1.00		
Middle	1.12	(0.91; 1.38)	1.25	(1.04; 1.50)	
High	1.68	(1.28; 2.21)	2.11	(1.78; 2.49)	
Attitude to violence					0.000
Low	1.00		1.00		
Middle	0.88	(0.55; 1.40)	1.09	(0.91; 1.30)	
High	0.95	(0.56; 1.59)	1.34	(0.92; 1.93)	
Decision-making					0.000
Low	1.00		1.00		
Middle	1.43	(1.13; 1.81)	1.28	(0.98; 1.67)	
High	1.87	(1.47; 2.36)	1.52	(1.20; 1.93)	
**Modern methods**					
Social independence					0.000
Low	1.00		1.00		
Middle	1.13	(0.95; 1.34)	1.02	(1.00; 1.04)	
High	1.38	(1.02; 1.85)	1.02	(0.96; 1.08)	
Attitude to violence					0.000
Low	1.00		1.00		
Middle	1.20	(1.09; 1.33)	1.03	(0.98; 1.08)	
High	1.29	(1.12; 1.49)	1.06	(1.01; 1.11)	
Decision-making					0.000
Low	1.00		1.00		
Middle	1.27	(1.14; 1.43)	1.10	(1.04; 1.16)	
High	1.45	(1.22; 1.73)	1.13	(1.05; 1.22)	

Sensitivity analysis

Supplementary Table 2. Demand for family planning satisfied according to religion, type of method, and women's empowerment in the Muslim-majority countries (Burkina Faso, Guinea, and Sierra Leone).

	Muslim		Christian	
	IRR	95% CI	IRR	95% CI
		**Traditional methods**		
Social independence				
Low	1.00		1.00	
Middle	0.84	(0.75; 0.93)	1.74	(0.72; 4.24)
High	1.24	(0.68; 2.27)	3.20	(1.27; 8.01)
Attitude to violence				
Low	1.00		1.00	
Middle	0.72	(0.47; 1.09)	1.51	(1.11; 2.05)
High	0.55	(0.37; 0.81)	2.07	(1.04; 4.15)
Decision-making				
Low	1.00		1.00	
Middle	1.42	(0.93; 2.15)	1.15	(0.67; 1.97)
High	1.67	(1.36; 2.06)	1.01	(0.68; 1.49)
		**Modern methods**		
Social independence				
Low	1.00		1.00	
Middle	1.02	(0.85; 1.23)	1.02	(0.89; 1.17)
High	1.26	(0.80; 1.99)	1.17	(0.81; 1.67)
Attitude to violence				
Low	1.00		1.00	
Middle	1.17	(0.98; 1.40)	1.13	(0.90; 1.42)
High	1.24	(1.11; 1.38)	1.17	(0.90; 1.52)
Decision-making				
Low	1.00		1	
Middle	1.23	(1.05; 1.44)	1.18	(0.94; 1.50)
High	1.30	(1.07; 1.57)	1.40	(1.16; 1.69)

Supplementary Table 3. Demand for family planning satisfied according to religion, type of method, and women's empowerment in the Christian-majority countries (Ethiopia, Ghana, Liberia, Malawi, Mozambique, and Uganda).

	Muslim		Christian	
	IRR	95% CI	IRR	95% CI
	**Traditional methods**			
Social independence				
Low	1.00		1.00	
Middle	1.31	(1.18; 1.45)	1.21	(0.90; 1.62)
High	2.69	(1.69; 4.29)	2.15	(1.57; 2.93)
Attitude to violence				
Low	1.00		1.00	
Middle	0.78	(0.55; 1.11)	1.25	(0.92; 1.68)
High	1.27	(0.89; 1.79)	1.31	(0.83; 2.06)
Decision-making				
Low	1.00		1.00	
Middle	1.15	(0.38; 3.53)	1.30	(1.03; 1.65)
High	1.21	(0.49; 2.94)	1.32	(0.96; 1.81)
	**Modern methods**			
Social independence				
Low	1.00		1.00	
Middle	1.09	(1.00; 1.18)	1.01	(0.99; 1.04)
High	1.04	(0.84; 1.28)	1.01	(0.96; 1.08)
Attitude to violence				
Low	1.00		1.00	
Middle	1.17	(1.05; 1.30)	1.03	(0.99; 1.06)
High	1.20	(1.12; 1.27)	1.05	(1.00; 1.09)
Decision-making				
Low	1.00		1.00	
Middle	1.17	(0.92; 1.48)	1.05	(1.03; 1.07)
High	1.39	(1.05; 1.83)	1.07	(1.02; 1.12)

Supplementary Table 4. Demand for family planning satisfied according to religion, type of method, and women's empowerment among the 40% poorest women.

	Muslim		Christian	
	IRR	95% CI	IRR	95% CI
	**Traditional methods**			
Social independence				
Low	1.00		1.00	
Middle	1.02	(0.75; 1.40)	1.06	(0.90; 1.26)
High	1.35	(0.91; 2.02)	1.37	(1.07; 1.76)
Attitude to violence				
Low	1.00		1.00	
Middle	1.02	(0.54; 1.92)	1.08	(0.79; 1.47)
High	0.71	(0.49; 1.04)	1.01	(0.61; 1.66)
Decision-making				
Low	1.00		1.00	
Middle	1.36	(0.89; 2.07)	1.42	(0.92; 2.18)
High	1.48	(1.04; 2.10)	1.67	(1.05; 2.66)
	**Modern methods**			
Social independence				
Low	1.00		1.00	
Middle	0.99	(0.84; 1.17)	0.98	(0.93; 1.04)
High	1.00	(0.87; 1.15)	0.95	(0.85; 1.05)
Attitude to violence				
Low	1.00		1.00	
Middle	1.12	(1.00; 1.25)	0.97	(0.91; 1.04)
High	1.21	(1.03; 1.43)	0.99	(0.93; 1.04)
Decision-making				
Low	1.00		1.00	
Middle	1.14	(1.02; 1.26)	1.05	(0.97; 1.14)
High	1.37	(1.14; 1.65)	1.07	(1.01; 1.14)

Supplementary Table 5. Demand for family planning satisfied according to religion, type of method, and women's empowerment among the 40% wealthiest women.

	Muslim		Christian	
	IRR	95% CI	IRR	95% CI
		**Traditional methods**		
Social independence				
Low	1.00		1.00	
Middle	1.05	(0.87; 1.26)	1.39	(1.13; 1.69)
High	1.46	(1.09; 1.96)	1.98	(1.74; 2.24)
Attitude to violence				
Low	1.00		1.00	
Middle	0.76	(0.51; 1.13)	0.89	(0.66; 1.21)
High	1.00	(0.57; 1.74)	1.11	(0.76; 1.62)
Decision-making				
Low	1.00		1.00	
Middle	1.36	(1.16; 1.59)	1.19	(0.97; 1.47)
High	1.75	(1.39; 2.19)	1.32	(1.06; 1.64)
		**Modern methods**		
Social independence				
Low	1.00		1.00	
Middle	1.07	(0.99; 1.16)	1.04	(0.98; 1.09)
High	1.18	(1.03; 1.34)	1.01	(0.95; 1.08)
Attitude to violence				
Low	1.00		1.00	
Middle	1.19	(1.05; 1.34)	1.03	(0.99; 1.08)
High	1.17	(1.15; 1.30)	1.06	(1.02; 1.10)
Decision-making				
Low	1.00		1.00	
Middle	1.25	(1.18; 1.32)	1.12	(1.05; 1.20)
High	1.25	(1.12; 1.40)	1.15	(1.04; 1.26)
